# Exploring the role of loop tunnels in enhancing termite food transport efficiency: A simulation approach

**DOI:** 10.1016/j.heliyon.2024.e28417

**Published:** 2024-03-19

**Authors:** Sang-Hee Lee, Cheol-Min Park

**Affiliations:** Division of Industrial Mathematics, National Institute for Mathematical Sciences, Daejeon, 34047, South Korea

**Keywords:** Termites, Food transport behavior, Traffic congestion, Individual-based model

## Abstract

Subterranean termites construct a network of tunnels beneath the ground, comprising a main tunnel and branch tunnels. While termites do not construct tunnels that turn back in a “U” shape, the intersection of main and branch tunnels often forms closed curved structures (a loop). The loop structure can have either a positive or negative effect on the transport efficiency of termites in food transportation. However, little research has been conducted on the impact of loop tunnels on transport efficiency (*E*) due to the technical difficulties associated with direct observation. In this study, we used an individual-based model to simulate termite behavior during food transportation within a tunnel composed of a main tunnel and a loop tunnel. The model incorporates four control variables: the number of introduced simulated termites (*k*_1_), the probability of a simulated termite using a loop tunnel when walking towards a food or nest site (*k*_2_ or *k*_3_), and the length of the loop tunnel (*k*_4_). The simulation results reveal that the *E* value is high for (*k*_2_, *k*_3_) = (high, high), (low, low), (high, low), and (low, high) when the length of the loop tunnel is relatively short. However, when the length of the loop tunnel is relatively long, *E* is high only for (*k*_2_, *k*_3_) = (high, low) and (low, high). We found that these results are primarily influenced by the frequency of traffic jams. Therefore, termites would benefit from adopting strategies that reduce the occurrence of traffic jams during food transportation. In the Discussion section, we briefly touch on the strategy from an ecological perspective.

## 1 Introduction

Subterranean termites, such as *Coptotermes formosanus* and *Coptotermes gestroi*, are known for their ability to construct tunnels underground in order to access food resources. These tunnels can span tens to hundreds of meters, requiring significant energy expenditure from the termites during construction [1,2]. Furthermore, termites should transport the food they discover back to their nests or subnests, resulting in additional energy consumption. Therefore, it is likely that they have developed strategies to minimize the cost of food transportation while maximizing the chances of finding food resources [3,4].

The foraging strategies of subterranean termites can be evaluated by their foraging efficiency, encompassing both searching efficiency and transport efficiency [4,5]. Understanding these efficiencies is crucial for comprehending the stability of termite systems. However, existing research has predominantly focused on search efficiency, as it can be assessed by creating termite tunnel patterns on a laboratory scale and subsequently conducting geometric and statistical analyses [6,7]. Several studies on searching efficiency have also indicated that termite tunnel patterns include a loop structure. It has been documented that termites tunnel radially from their initial site, avoiding the formation of a loop-back “U” tunnel in a homogeneous sandy substrate [8]. However, radially extended tunnels may intersect, leading to the formation of loopback tunnels. Additionally, external environmental conditions can exert forces that may cause the tunnel to bend, further contributing to the creation of loopback tunnels.

Puche and Su (2001) [9] observed that termites (*Reticulitermes flavipes* and *Coptotermes formosanus*) tunneling in two-dimensional sandy substrates displayed longer main tunnels and a greater number of branch tunnels when the sand contained higher moisture levels. Notably, the tunneling pattern exhibited a fractal structure. Fractal patterns are commonly observed in the flow of matter within diverse systems, such as plant roots, river channels, and blood vessels within the human body. These patterns are known to be efficient structures for material flow and movement [10,11], indicating that termites construct tunnels optimized for food transportation. The fractal pattern of tunnels shows that the intersection of tunnels creates loop structures of different sizes. Arab and Costa-Leonardo (2005) [12] also noted that the total area of tunnels constructed by two termite species, *Coptotermes gestroi* and *Heterotermes tenuis*, increased with rising temperatures. However, the presence of food did not impact tunneling in *C. gestroi*, whereas it led to a reduction in the number of tunnels formed by *H. tenuis*. Cornelius and Osbrink (2010) [13] observed that the physical properties of soil, such as particle size and moisture level, exert influence over the tunneling behavior and shelter tube structure of termites. Their findings demonstrated that these influences resulted in shelter tube patterns that prominently featured loop structures. Additionally, Lima and Costa-Leonardo (2012) [7] demonstrated that physical guidance resulted in an extension of primary tunnel length and a decrease in branching frequency in *C. gestroi* termites. Bardunias and Su (2010) [14] revealed that termite tunneling patterns are determined by two distinct termite behaviors. One behavior involves excavating sidewalls to create branching tunnels, while the other involves waiting for existing tunnels to expand. The frequency of these two behaviors was found to play a crucial role in the formation of diverse tunnel patterns.

In addition to the previously mentioned studies, there exist experimental findings that offer a more nuanced understanding of food transport efficiency. For example, research suggests that older workers tend to be located further from the nest [15]. Moreover, termite colonies typically maintain a soldier population of approximately 10%, which influences worker behavior [16]. It has been observed that a significant portion, roughly 70–80%, of workers remain within the nest without actively engaging in food transport [15,17]. In a natural termite system, the extent of individuals involved in the food search and transport process depends on the food quantity available in the soil [18]. Another crucial example is the mechanism of tunnel branching. Termites encountering depressions in tunnel walls may either excavate soil to form new branches or deposit soil to fill existing voids. The likelihood of branching is contingent upon the equilibrium between soil deposition to fill voids and excavation to expand them [19].

In contrast to the aforementioned studies on search efficiency, there have been very few investigations into food transport efficiency due to several technical challenges. Observing termite movement within tunnels is difficult due to the uneven distribution of individuals. Even when artificial tunnels are utilized, tracking and observing individual termites remain challenging [20,21]. Additionally, variable tunnel widths [22] and varying walking speeds of individuals [23] further complicate matters. Furthermore, the specific mechanisms (modalities) of termite transport behavior are not yet clearly understood. As there have been no experimental studies on termite food transportation to date, researchers have proposed three potential modes of transportation inspired by the methods ants use: (1) direct carrying of food from the feeding site to the nest by individual termites, (2) transferring food particles to other termites for subsequent transport to the nest (bucket brigade mode), and (3) depositing food particles at specific locations along the feeding route for other termites to collect and carry to the nest. Anderson et al. (2002) [24] suggested that the bucket brigade mode would be advantageous in narrow, elongated routes connecting feeding sites and nests. Considering that subterranean termite tunnels are lengthy (tens of meters) [1,2] and relatively narrow (4–5 mm), it is likely that the bucket brigade mode is the preferred method of food transport [3].

In a previous study [25], an individual-based model was developed to simulate the food transport behavior of termites within a sinusoidally shaped tunnel, assuming a bucket brigade mode. The model aimed to investigate the impact of variables such as the probability of food transfer between individuals, the number of individuals involved in food transfer, the variation in walking speed of individuals with tunnel curvature, and the loss of food during transfer on food transport efficiency. In a subsequent study [26], the same model was utilized to examine the influence of tunnel surface irregularities on food transport efficiency. These two studies indirectly demonstrated that the frequency of traffic jams caused by food-carrying termites could be a crucial factor in determining food transport efficiency. However, the analysis of the traffic jam effect was limited by the constraints of the model, which only considered short tunnel sections and did not accurately reflect the structural characteristics of actual termite tunnels, such as path connectivity for tunnels.

In this study, we developed a model that considers the elongated length of real termite tunnels and the loop tunnels formed by the intersection of main and branch tunnels.

### 1.1 Model description and analysis

#### 1.1.1 Individual-based model

In this study, we have developed an individual-based model that simulates the process of food transport by termites, from a feeding site to their nest. The model focuses solely on the termites’ participation in the food transport process, without considering the utilization of chemical cues or the construction of new tunnels. Additionally, the model only takes into account worker termites, disregarding the presence of soldier termites. Moreover, it assumes equal activity and dedication to food transport among all simulated termites. However, these assumptions oversimplify the complexity of actual termite systems.

For our simulations in this study, we conducted a total of 20 simulations for each of the four control variables: the number of participating termites (*k*_1_), the probability of a simulated termite using a loop tunnel when heading to a food source or nest (*k*_2_ or *k*_3_), and the length of the loop tunnel (*k*_4_). Each simulation had a duration of 10,000 time steps.

#### 1.1.2 Termite main and loop tunnel

**Fig. 1:**
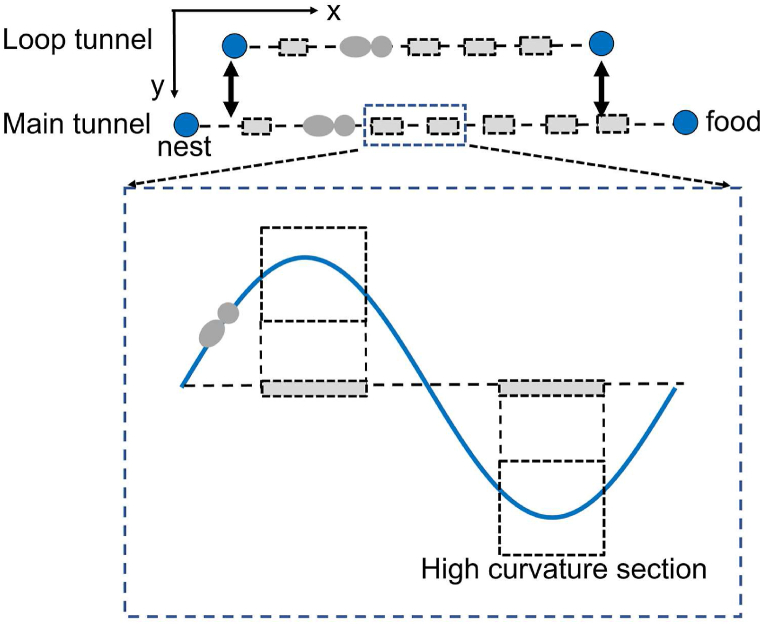
(Top) A schematic representation of the main tunnel and its connected loop tunnel. The dotted gray squares indicate tunnel sections with high curvature. The ends of the main tunnel serve as nesting and food sites. (Bottom) A magnified view of two selected gray squares within the main tunnel, demonstrating a single sinusoidal-shaped tunnel segment with one period.

We have constructed a tunnel consisting of 500 grid cells, spanning from (1, 250) to (500, 250), within a 500 × 500 grid space (top of Fig. 1). The endpoint (1, 250) represents the nest site, while the endpoint (500, 250) represents the foraging site. This tunnel is referred to as the main tunnel. The sections with high curvature are depicted as gray dotted squares. In our model, each gray square corresponds to a single grid cell located at *x* = 10, 63, 117, …, 490. The simulated tunnel exhibits two high curvature sections, forming a sinusoidal-shaped tunnel with one period (see the bottom of Fig. 1).

Additionally, we have created another tunnel comprising grid cells from (10, 125) to (10+(*k*_4_-1) × 48, 125). This tunnel is referred to as the loop tunnel. The term “loop tunnel” might be potentially misleading in this context. While actual termites do not construct tunnels that turn back in a “U” shape, a geometric loop structure can emerge when tunnels radiating from the tunneling site intersect. In this study, we define “loop tunnels” as such geometric formations. The location of the high curvature section in the loop tunnel coincides with the location of the high curvature section in the main tunnel (in terms of *x* coordinates). As *k*_4_ increases, the length of the loop tunnel also increases. To ensure that the simulated termites do not immediately encounter the junction of the main and loop tunnels upon leaving the food site, the loop tunnel starts at the grid cell (10, 125).

When a simulated termite travels along the main tunnel towards the food site, there is a probability of jumping into the loop tunnel at *x* = 10, denoted by *k*_2_. The jumping probability is given by (*k*_2_-1) × 0.1, where *k*_2_ ranges from 1 to 11. When *k*_2_ = 1, all simulated termites traveling towards the food site refrain from jumping into the loop tunnel. Conversely, when *k*_2_ = 11, all simulated termites traveling towards the food site follow the main tunnel. The process of jumping does not involve any time spent by the simulated termites. Similarly, when a simulated termite faces the nest, it decides whether to jump into the loop tunnel at grid coordinates *x* = (10, 125). The jumping probability is determined by *k*_3_ and is given by (*k*_3_ -1) × 0.1.

#### 1.1.3 Behavior of simulated termites

**Fig. 2:**
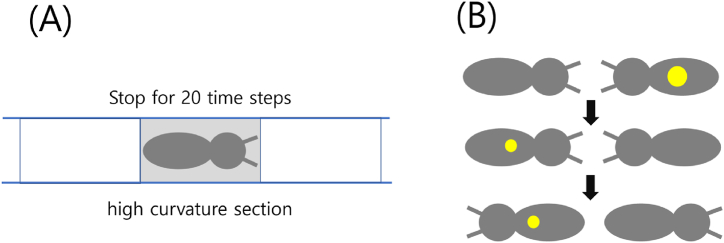
(A) An illustration of the stopping behavior of a simulated termite upon entering the highly curved tunnel depicted by the gray square. (B) A visual representation of the encounter and food particle transfer between two simulated termites. The yellow circle represents food, while the small circles indicate food loss. (For interpretation of the references to colour in this figure legend, the reader is referred to the Web version of this article.)

To simulate the food transport behavior observed in real termites, we have imposed several behavioral rules on the simulated termites in our model. When a simulated termite encounters a grid cell with high curvature, it pauses for 20 time steps (Fig. 2A). This rule is based on observations by Refs. [27,28], which indicate that real termites reduce their walking speed when traversing curved sections. It is important to note that the value of 20 time steps does not represent the actual walking speed of termites, but rather a carefully chosen value to simulate slow walking.

When a simulated termite carrying a food particle encounters another termite without food, a food transfer event occurs with a probability of 0.001 (Fig. 2B). All simulated termites are programmed to pick up one food particle at a time from the food site, with each particle's quantity set to 1.0. To account for food loss during the transfer process, we have incorporated a rule of 0.005 loss per transfer. Therefore, if a food particle is successfully conveyed to the nest with a single loss event, the amount of transported food would be 0.995. In the figure, the yellow circles on the two simulated termites represent food particles. The smaller size of the yellow circles after the food has been transferred indicates food loss. If multiple losses occur, causing the quantity of food particles to drop below zero, the amount of food particles is considered zero. In actual termites, food transfer can occur through stomodeal and proctodeal trophallaxis. If termites employ proctodeal trophallaxis for food transfer, traffic jams may aid in the transfer of food from one termite's anus to the mouths of other termites. However, the model does not distinguish between the two modes of food transfer, but only characterizes food losses. When two simulated termites engage in food transfer, they are allowed to change their direction. If no food transfer occurs, the two termites simply pass each other without physical interaction. The transport efficiency, *E*, was defined as follows:

(1) $$E\left(\tau \right)=\left(\frac{s\times \sum_{t=1}^{\tau }food\left(t\right)}{\tau \times N_{0}}\right)$$

Here, the variable “food(*t*)” denotes the quantity of food particles that the simulated termites have gathered in the nest at time *t*. The variable *N*_0_ represents the count of termites involved in the process of transporting food. Furthermore, the constant s is assigned a value of 10,000 to appropriately amplify *E*, which would otherwise have a value that is too small.

For our simulation model, we initially place *N*_0_ (=30, 51, 72, …, 240) simulated termites randomly within the tunnel at *t* = 0. None of these initially introduced simulated termites are provided with any food particles. To maintain simplicity and consistency in representing variable values, we introduce four control variables: *k*_1_, *k*_2_, *k*_3_, and *k*_4_, each ranging from 1 to 11. The relationship between *N*_0_ and *k*_1_ is defined as *N*_0_ = 30 + (*k*_1_-1) × 21. Further details regarding the remaining parameters are provided in subsequent sections.

#### 1.1.4 Traffic jam

**Fig. 3:**
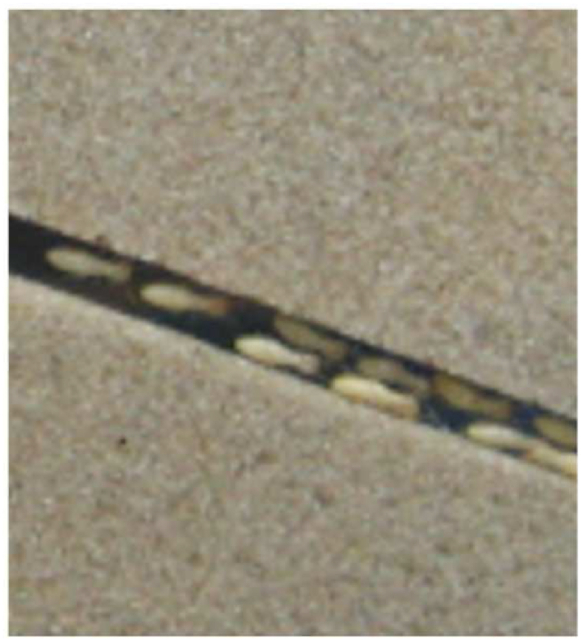
Traffic jamming behavior observed in termites passing through an artificial tunnel. The tunnel, measuring 3 mm in width and 100 mm in length, is created between two acrylic panels filled with sand.

As mentioned briefly in previous studies [27,28], we created artificial tunnels within a substrate filled with uniform sand and placed termites inside them to observe traffic jamming, albeit without statistical analysis. The tunnels had a length of 100 mm and a width of either 2 or 3 mm, and we introduced 10 termites into each tunnel. Some termites walked through the tunnel, paused, and then reversed their direction to continue, while others maintained their initial direction. Certain termites halted when they encountered other individuals, and this stopping behavior sometimes resulted in subsequent stoppages of the following termites (Fig. 3). We refer to this phenomenon as “traffic jams.”

In our model, a traffic jam is assumed to occur when two conditions are met: (1) four or more simulated termites are located in the same grid cell, and (2) the sum of the termite walking direction vectors is zero. However, we do not delve into the specific details of traffic jams in this study. Simulated termites trapped in a traffic jam remain motionless and do not interact with other individuals for 100 time steps. After this duration, they resume walking in the direction they were traveling before coming to a stop.

## 2 Results

**Fig. 4:**
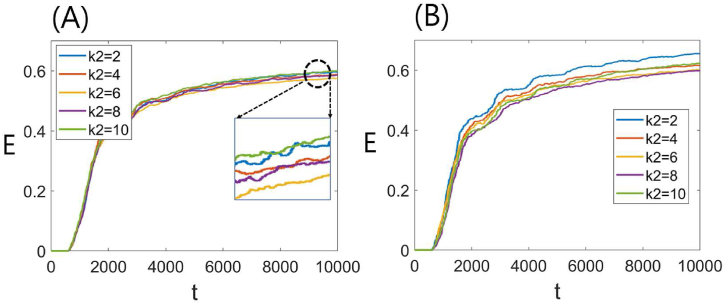
(A) Plot of food transport efficiency (*E*) against time. Here, *k*_2_ takes values from 2 to 10, while *k*_1_, *k*_3_, and *k*_4_ are fixed at 7, 6, and 3, respectively. The inset provides a magnified view of the dotted circle. (B) *E* as a function of time. The graph represents varying values of *k*_2_ from 2 to 10, with fixed values of *k*_1_ = 7, *k*_3_ = 10, and *k*_4_ = 3.

We set *k*_1_ and *k*_4_ as 7 and 3, respectively, to investigate the impact of *k*_2_ and *k*_3_ on the transport efficiency, *E* (see Eq. (1)). Fig. 4 illustrates the temporal dynamics of the *E* value. Initially (*t* = 0), all simulated termites were introduced without food particles, resulting in an *E* value of zero in the range *t* < 500. This implies that even the simulated termite closest to the food site requires a minimum of 501 time steps to transport the food particles to the nest. In the range 500 ≤ *t* < 2000, the initially introduced simulated termites sequentially picked up food particles from the food site and reached the nest, causing the *E* value to increase rapidly. As more simulated termites made their way to the nest, the *E* value exhibited a slow approach towards saturation in the *t* > 2000 region. The inset figure (Fig. 4A) magnifies the saturation region (dashed circle). Increasing *k*_2_ led to a decrease in *E*, followed by a slight increase. For *k*_2_ = 10 (Fig. 4B), the *E* value displayed a similar pattern to Fig. 4A, albeit with a slightly higher overall value. The gap between the *E* curves widened with each *k*_2_ value, indicating that *k*_3_ slightly augmented the effect of *k*_2_ on *E*.

**Fig. 5:**
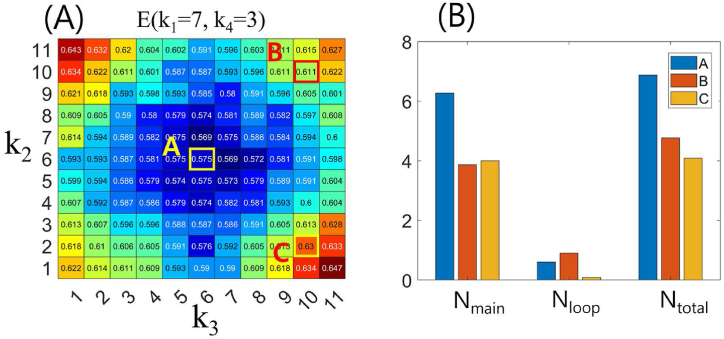
(A) Plot of *E*(*k*_1_ = 7, *k*_4_ = 3) as a function of *k*_2_ and *k*_3_. The points A, B, and C represent the values (*k*_2_, *k*_3_) = (6, 6), (10, 10), and (2, 10), respectively. (B) Frequency of traffic jams in the main tunnel (*N*_main_), loop tunnel (*N*_loop_), and the entire tunnel (*N*_total_) for conditions A, B, and C.

Fig. 5 illustrates the variation in transport efficiency *E* when the simulated termites utilized the loop tunnel in conjunction with the main tunnel. Specifically, we analyzed the *E*(*k*_1_, *k*_2_, *k*_3_, *k*_4_) map for *k*_1_ = 7 and *k*_4_ = 3 to investigate the influence of *k*_2_ and *k*_3_ on *E* (Fig. 5A). The *E* maps exhibit an overall symmetric tendency for *k*_2_ and *k*_3_, implying that simulated termites traveling to the food site and those traveling to the nest site behave symmetrically, despite some symmetry disruption caused by traffic jams. Interestingly, the minimum *E* value was observed at (*k*_2_, *k*_3_) = (6, 6), indicating that E is at its lowest when the simulated termites use the main and loop tunnels with equal probabilities of 0.5 each. The *E* map reveals higher *E* values in the four corner regions. To comprehend this simulation outcome, we assessed the frequency of traffic jams in the main and loop tunnels for (*k*_2_, *k*_3_) = (6, 6), (10, 10), and (2, 10) on the *E* map (Fig. 5B). Each condition was labeled as *A*, *B*, and *C*, respectively. *N*_main_ represents the frequency of traffic jams in the main tunnel, *N*_loop_ represents the frequency of traffic jams in the loop tunnel, both divided by 100,000. The total frequency of traffic jams is denoted as *N*_total_. At site *A*, where (*k*_2_, *k*_3_) = (6, 6) and the minimum *E* value occurs, *N*_main_ is high and *N*_loop_ is relatively low. At site *B*, where (*k*_2_, *k*_3_) = (10, 10), *N*_main_ sharply decreases, while *N*_loop_ experiences a slight increase. At site *C*, where (*k*_2_, *k*_3_) = (2, 10), *N*_main_ slightly increases, whereas *N*_loop_ decreases significantly. These results indicate that simulated termites traveling to food and nest sites enhance *E* when they have a high probability of choosing between tunnels or loop tunnels, or both with high probability. *E* values were also high when loop tunnels were used with a low probability.

**Fig. 6:**
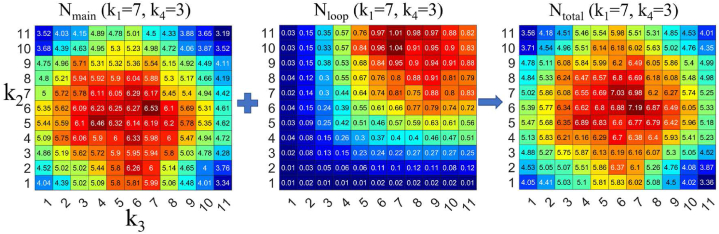
(Left) *N*_main_(*k*_1_ = 7, *k*_4_ = 3) plotted against *k*_2_ and *k*_3_. (Middle) *N*_loop_(*k*_1_ = 7, *k*_4_ = 3) plotted against *k*_2_ and *k*_3_. (Right) *N*_total_(*k*_1_ = 7, *k*_4_ = 3), which represents the sum of *N*_main_ and *N*_loop_, plotted against *k*_2_ and *k*_3_.

To confirm the results presented in Fig. 6 for the entire *E*(*k*_2_, *k*_3_) map, we examined *N*_main_ and *N*_loop_ for *E*(*k*_2_, *k*_3_) where *k*_1_ = 7 and *k*_4_ = 3 (Fig. 6). The jamming frequency in the main tunnel, *N*_main_, tends to increase dramatically towards the center of the *E*(*k*_2_, *k*_3_) map. As the jamming frequency increases, the *E* value decreases, which explains why the *E* value is minimal at (*k*_2_, *k*_3_) = (6, 6). On the other hand, *N*_loop_ increased as *k*_2_ and *k*_3_ increased. This trend is quite different from that of *N*_main_, but since the *N*_loop_ values were relatively small compared to the *N*_main_ values, *N*_total_ was determined by *N*_main_.

**Fig. 7:**
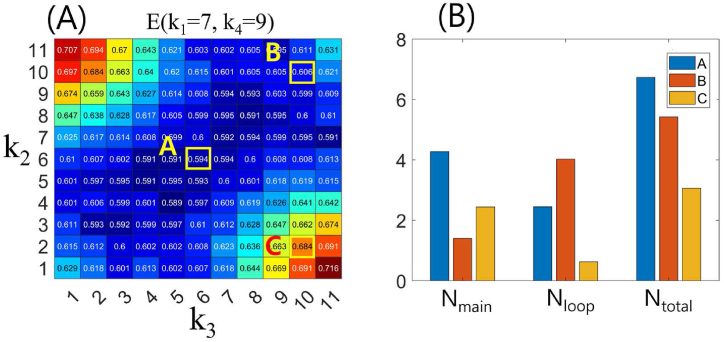
(A) Plot of *E*(*k*_1_ = 7, *k*_4_ = 9) as a function of *k*_2_ and *k*_3_. The points A, B, and C represent the values (*k*_2_, *k*_3_) = (6, 6), (10, 10), and (2, 10), respectively. (B) Frequency of traffic jams in the main tunnel (*N*_main_), loop tunnel (*N*_loop_), and the entire tunnel (*N*_total_) for conditions A, B, and C.

To investigate the effect of *k*_4_ on the *E* map presented in Fig. 5, we examined the *E* map for *E*(*k*_2_, *k*_3_) where *k*_1_ = 7 and *k*_4_ = 9 (Fig. 7). In this investigation, we found that *E* values were high when one of *k*_2_ and *k*_3_ was large and the other was small, or vice versa (Fig. 7A). To comprehend this simulation outcome, we assessed the frequency of traffic jams in the main and loop tunnels for (*k*_2_, *k*_3_) = (6, 6), (10, 10), and (2, 10) on the *E* map (Fig. 7B). This is because increasing the length of the loop tunnel (*k*_4_) decreased the total simulated termite flow density due to the loop tunnel. This means that the *E* value is relatively high when the simulated termites primarily choose to use either the main tunnel or the loop tunnel. The frequency of traffic jams at the three locations *A*, *B*, and *C* on the *E*(*k*_2_, *k*_3_) map resulted in a relatively higher *E* value when *k*_4_ = 9 than when *k*_4_ = 3, and the *N*_loop_ value was much higher when *k*_4_ = 9.

**Fig. 8:**
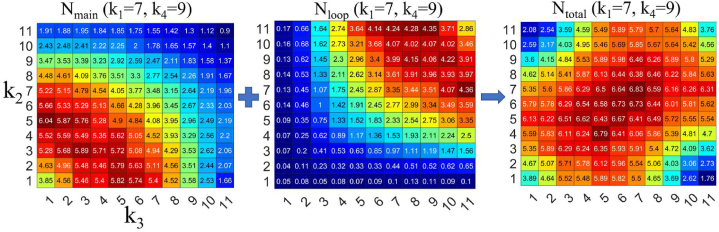
(Left) *N*_main_(*k*_1_ = 7, *k*_4_ = 9) plotted against *k*_2_ and *k*_3_. (Middle) *N*_loop_(*k*_1_ = 7, *k*_4_ = 9) plotted against *k*_2_ and *k*_3_. (Right) *N*_total_(*k*_1_ = 7, *k*_4_ = 9), which represents the sum of *N*_main_ and *N*_loop_, plotted against *k*_2_ and *k*_3_.

To gain a better understanding of the *E* map shown in Fig. 7, we examined the frequency of traffic jams caused by the simulated termites for the main and loop tunnels under the condition of *k*_1_ = 7 and *k*_4_ = 9 (Fig. 8). *N*_main_ increased as *k*_2_ and *k*_3_ became smaller together and decreased as *k*_2_ and *k*_3_ became larger together. *N*_loop_ followed the reverse trend. This is because the influence of the loop tunnel on *E* increased as the length of the loop tunnel, *k*_4_, became comparable to the length of the main tunnel. Therefore, *N*_total_, the sum of *N*_main_ and *N*_loop_, showed a different trend from the results in Fig. 6.

**Fig. 9:**
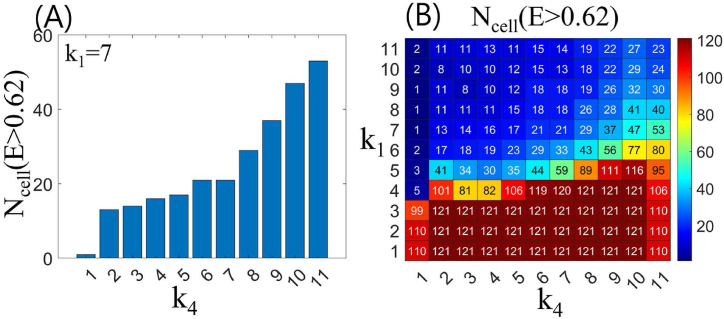
(A) Number of cells, *N*_cell_, in *E*(*k*_2_, *k*_3_) exceeding 0.62, with *k*_1_ = 7 and *k*_4_ = 1, 2, …, 11. (B) Map displaying the distribution of *N*_cell_ for *k*_1_ and *k*_4_ values ranging from 1 to 11.

To investigate the effect of *k*_4_ on *E* as *k*_1_ varies, we counted the number of cells, *N*_cell_, in the *E*(*k*_2_, *k*_3_, *k*_4_) with *k*_1_ = 7, where the value of each grid cell is greater than 0.62 (Fig. 9). A larger *N*_cell_ indicates a larger area of high *E* values in the *E*(*k*_2_, *k*_3_) map. The value of 0.62 is chosen appropriately by examining the entire dataset. When *k*_1_ = 1, *N*_cell_ was close to zero and slowly increased in the range 2 ≤ *k*_4_ ≤ 7, and then sharply increased at *k*_4_ > 7 (Fig. 9A). These results indicate that the presence of loop tunnels, even if the length of the loop tunnel is short, positively contributes to the improvement of termite transport efficiency. Furthermore, we found that the effect of *k*_4_ on *E* is divided into two parts, with relatively large and small effects. This result holds regardless of the value of *k*_1_, but the effect of *k*_4_ on *E* tends to gradually decrease as *k*_1_ increases (see the boundaries of the red and blue regions in Fig. 9B).

## 3 Discussion

Subterranean termite, such as *Coptotermes formosanus* and *Coptotermes gestroi*, tunnels typically consist of a main tunnel and a branch tunnel that intersect to form a loop structure. In this study, we refer to this structure as a loop tunnel. Loop tunnels provide termites with multiple routes to reach their destination, suggesting a significant impact on termite traffic flow within the tunnels, which is closely related to food transport efficiency. However, to our knowledge, there is limited research on this aspect. Therefore, we aimed to investigate how loop tunnels affect food transport efficiency by examining the frequency of traffic jams in our simulation approach.

The simulation results show that for smaller values of *k*_4_, higher efficiencies are observed in the four-corner region of the *E*(*k*_2_, *k*_3_) map (Fig. 5). On the other hand, for larger values of *k*_4_, the transport efficiency is higher when either *k*_2_ or *k*_3_ has a high value and the other has a low value in the *E*(*k*_2_, *k*_3_) map (Fig. 7). This result is in good agreement with the frequency distribution of traffic jams observed in the main and loop tunnels (Fig. 6, Fig. 8).

**Fig. 10:**
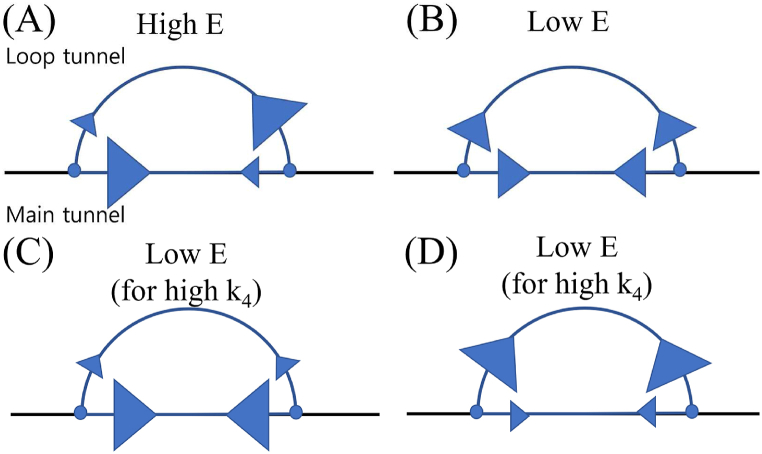
Various scenarios illustrating the probability of simulated termites utilizing the main and loop tunnels. The larger triangles correspond to higher probability values, while the smaller triangles represent lower probability values. (A) represents the case with high *E*, while (B), (C), and (D) represent the cases with low *E*.

The results suggest that reducing the frequency of traffic jams is a viable strategy for termites to enhance their food transport efficiency. One such strategy is to utilize different tunnel paths, particularly when the simulated termites move in distinct directions. To illustrate this way, we present four examples of simulated termite path selection, where the size of the triangles represents the associated probability value for each direction (Fig. 10). Among these examples, the upper left case, where simulated termites moving in different directions follow separate paths, demonstrates the highest food transport efficiency.

**Fig. 11:**
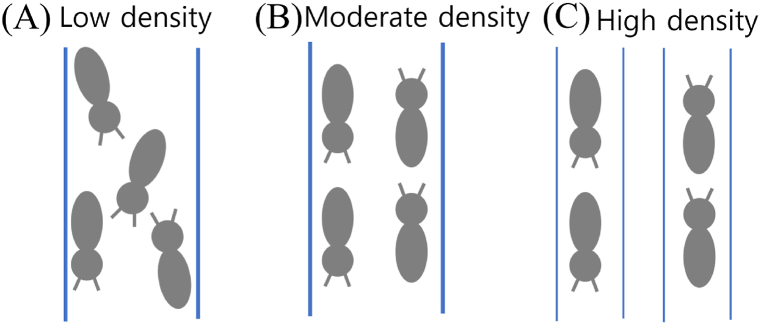
Illustration depicting termite traveling behavior under varying termite densities within the tunnel, including (A) low density (free-running), (B) moderate density (forming lanes), and (C) high density (creating loop tunnels).

Based on this strategy, the following inferences can be drawn (Fig. 11): at low termite densities within the tunnel, individuals traveling in different directions exhibit somewhat disorganized movement; at moderately high densities, bidirectional lanes are formed within the tunnel; and at high densities, employing different tunnels for distinct flow directions may enhance food transport efficiency. As a related study to our inference, Hedlund and Henderson (1999) [29] investigated the impact of food size on the volume and length of tunnels in the Formosan subterranean termite, *Coptotermes formosanus* Shiraki. However, Campora and Grace (2001) [30] concluded that the presence of food did not influence the distribution of tunnels in *C. formosanus*. Similarly, Puche and Su (2001) [31] reported that the tunnel geometry of *C. formosanus* in a laboratory arena remained unaffected by the presence of food. These experimental studies primarily focused on analyzing basic geometric characteristics such as tunnel length, width, and quantity, which do not directly address our proposed inference. To test our inference, it would be worthwhile to compare the number of loop tunnels near and far from food under different population densities. This topic is left as a potential area for future research.

While the model used in this study incorporates important variables related to termite food transport, it does possess certain limitations. Particularly, the model does not account for the statistically slower transit time of termites in wider tunnels, as observed in experiments [23]. This experimental observation is not reflected in the model. Furthermore, the model only considers a single loop tunnel, which oversimplifies the real-world scenario. Therefore, it is necessary to consider multiple loop tunnels of varying lengths. Despite these limitations, this study holds value not only because it undertakes a theoretical exploration of termite transport efficiency, an area that has received little attention, but also because it suggests potential strategies that termites may employ to enhance food transport efficiency.

## Data availability statement

No data was used for the research described in the article. No data associated with this study has been deposited into a publicly available repository.

## Additional information

No additional information is available for this paper.

## CRediT authorship contribution statement

**Sang-Hee Lee:** Writing – review & editing, Writing – original draft, Methodology, Investigation, Data curation, Conceptualization. **Cheol-Min Park:** Writing – review & editing, Validation, Investigation.

## Declaration of competing interest

The authors declare that they have no known competing financial interests or personal relationships that could have appeared to influence the work reported in this paper.
